# Severe postpartum disruption of the pelvic ring: report of two cases and review of the literature

**DOI:** 10.1186/1754-9493-5-2

**Published:** 2011-01-13

**Authors:** Zhiyong Hou, John T Riehl, Wade R Smith, Kent A Strohecker, Patrick J Maloney

**Affiliations:** 1 Department of Orthopaedics, 3rd Hospital, Hebei Medical University, Shijiazhuang, Hebei 050051, PR China; 2 Department of Orthopaedic Surgery, Geisinger Clinic, 100N. Academy Ave, Danville, PA 17822, USA

## Abstract

Pelvic dislocations are rare during labor, and the treatment is controversial. We report two cases of young women who sustained postpartum disruption of the pelvic ring: one case is an 8.8 cm wide separation of the pubic symphysis with sacroiliac joint disruption underwent surgical stabilization and the second case with 4.0 cm disruption being treated non-operatively. These cases illustrated of importance of accurate diagnosis, careful physical exam, fully informed consent and specific treatment for this condition.

## Background

Disruption of the symphysis pubis is a rare injury during childbirth with an incidence of 0.005% to 0.8% [1-3]. It is usually seen in elderly (older than 35) primagravida and there are few reports of this injury in younger patients [4,5]. Conservative treatment was reported to be successful in most cases, and operative management was considered in severe displaced case. We report two cases of young women who sustained postpartum disruption of the pelvic ring: one case is an 8.8 cm wide separation of the pubic symphysis with sacroiliac joint disruption underwent surgical stabilization and the second case with 4.0 cm disruption being treated non-operatively. These cases illustrated of importance of accurate diagnosis, careful physical exam, fully informed consent and specific treatment for this condition.

## Case presentation

### Case 1

**Figure 1:**
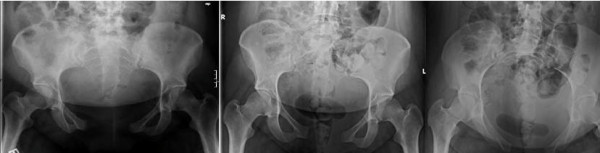
**X-rays showing the symphysis pubis with 8.8 cm diastasis initially and 6.8 cm diastasis left with bilateral sacroiliac joint widening after one week of bed rest**.

**Figure 2:**
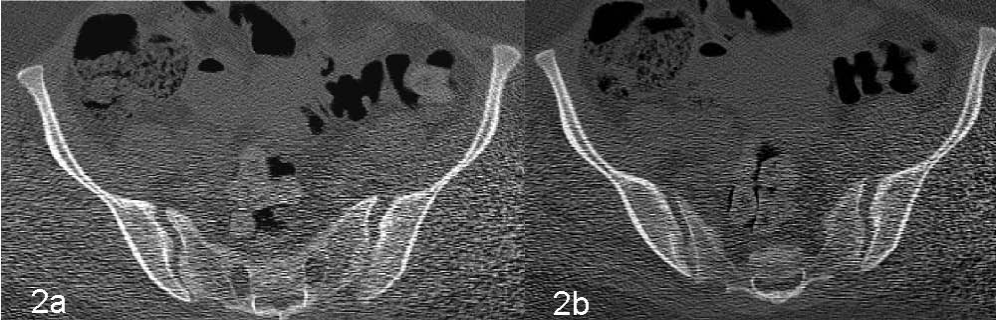
**CT scan showing widening of bilateral sacroiliac joints with a slight subluxation of left sacroiliac joint**.

A 26-year-old female patient had a history of two previous uncomplicated pregnancies 4 and 7 years prior. She underwent a vaginal delivery, delivering a healthy macrosomic fetus with a vacuum assistance at an outside hospital. The patient labored for 50 minutes and received epidural anesthesia for pain control peripartum. Immediately after delivering a healthy male (9 Lb 42 oz) she developed sudden severe pain over the pubic symphysis and buttocks. She also complained of left lower extremity weakness. On exam, tenderness in the symphysis pubis and the left sacroiliac joint were elicited. Radiographs revealed an 8.8 cm diastasis of the pubic symphysis (Figure 1). CT showed widening (anteriorly) of the sacroiliac joints, bilaterally with slight posterior subluxation of the left sacroiliac joint (Figure 2). The treating orthopaedic surgeon called to our regional trauma center for consultation. We advised bed rest and image recheck in one week. After one week of bed rest with the hope for closure of the defect on its own, the diastasis at the pubic symphysis remained with 6.8 cm separation, and the patient was referred to our institution for further evaluation one week after delivery.

**Figure 3:**
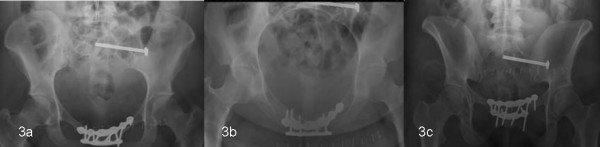
**Symphysis pubis and sacroiliac joint reduced and fixed by two orthogonal plates and one sacroiliac screw**.

The patient was painful anterior and posterior with an apparent APC III pelvic injury, surgical reduction and stabilization was recommended. Because of the extensive soft tissue stripping, small bones, and patients need for earlier full weight bearing to take care of children, two orthogonal plates were placed on the superior and anterior surfaces. The sacroiliac joints were then examined with fluoroscopy and slight posterior subluxation was found on the left side. A sacroiliac screw was placed to reduce and stabilize the left sacroiliac joint (Figure 3).

Two weeks after operation the patient's wound healed, and her pain had subsided. The patient insisted on walking with full weight bearing on both legs in order to care for her children. She complained of low back and anterior pelvic pain with activities of daily living until 4 months. The patient's recovery is excellent, with no evidence of the previous complaints with 12 months follow up.

### Case 2

**Figure 4:**
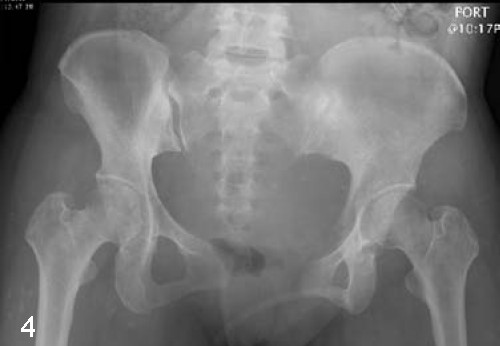
**X-rays showing the symphysis pubis with 4.0 cm diastasis initially**.

**Figure 5:**
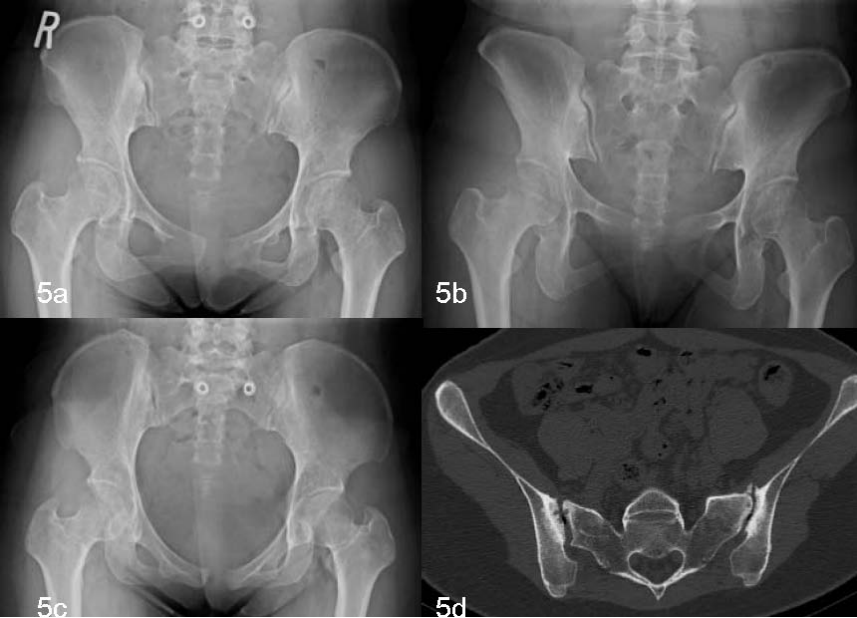
**X-rays showing pelvic diastasis disruption reduced to fifteen millimeters**. CT showing the right sacroiliac joint was not widened but associated with sclerosis consistent with sacroiliitis.

The patient is a 29-year-old female (gravida III, para III) referred for evaluation of pain and pubic diastasis 2 months after her last delivery. Immediately after delivery, the patient complained of significant anterior pain and extreme difficulty with weight bearing and ambulatory status post her delivery. Clinical and radiologic diagnosis confirmed a 40 mm diastasis of the symphysis pubis. On the first AP roentgenogram of the pelvis, a 40 mm separation of the symphysis and widening of the sacroiliac joint were seen (Figure 4). The patient was advised at an outside hospital to maintain bed rest and wear a pelvic binder. After 2 months, she still had significant pain in her right posterior sacroiliac area and her anterior superior iliac area. This improved somewhat but she was almost unable to ambulate. When she presented for evaluation in our clinic, she had had a positive FABER test, equal leg lengths and significant pain to palpation. After pelvic diastasis disruption was reduced from 40 mm down to 15 mm on the AP pelvic roentgenogram, the right sacroiliac joint was not widened but associated with sclerosis consistent with sacroiliitis on CT (Figure 5).

After lengthy informed consent, the patient chose continued non-operative management. Ten months after injury, she is mildly painful in her right posterior sacroiliac region, ambulates with a slight limp but is handling activities of daily living without issue.

## Discussion

The extent of symphyseal changes during pregnancy and delivery may vary significantly. Peripartum ligamentous relaxation with moderate widening of symphysis pubis and sacroiliac joint is physiologic and occurs regularly, which is thought to be hormonally mediated by relaxin and progesterone [6]. Ligamentous relaxation provides relative mobility of the pubic symphysis and SI joint synchondroses, resulting in widening of the birth canal and facilitating delivery. After delivery, laxity of these ligaments gradually diminishes, the pubic diastasis disappears, and pelvic stability is restored.

A displaced injury with diastasis 8.8 cm is rare (Table 1). Kharrazi et al reported four cases with the average pubic symphysis diastasis of 6.4 cm (range: 6.1-6.6). Physiologic peripartum symphyseal widening ranges from 3 to 7 mm and often remains asymptomatic [7]. Slight pubic diastasis in the absence of clinical symptoms is frequent and does not necessitate medical treatment. Most investigators have stated that separation of more than 1 cm is pathologic with concomitant rupture of the four symphyseal ligaments and renders the pubic symphysis unstable [8,9]. Symphysiolysis is seldom associated with diastasis of more than 1.5 cm [7]. Pauwels considered that significant separation of the anterior ring is needed to damage the posterior components of the pelvis, and a diastasis of over 2.5 cm means that the sacroiliac ligaments have been damaged and operative treatment should be recommended [5]. If symphyseal separation exceeds 4 cm, the sacroiliac joints may be damaged as well, and some cases are accompanied with lumbosacral plexopathy due to dislocation [7,8].

**Table 1:** Case series of Postpartum Disruption of the Pelvic Ring in recent year.

Author/s (year)	cases	pregnancy	symphysis diastasis	SI	treatment	follow-up
Dhar(1992)^9^	2	Second	2.3cm;1.8cm		Bed rest	Normal after 3 months

Pennig (1997)^3^	1	Second	3 cm	Right widening	external fixator	normal
Kharrazi (1997)^7^	4	3/4 second	Average initial 6.4 cm(range: 6.1-6.6)	sclerosis	Bed rest and pelvic binder	All residual pain and disability
Rommens(1997)^12^	3	2 first; 1 third	1.5 cm, after 4-6 weeks of bed rest		Symphysis plate and one SI screw	All free of complaints
Hierholzer(2007)^13^	1	Second	9 cm	Both widening	Symphysis plate and both SI screw	Recovery after 23 months,

The mechanism of postpartum pelvic disruption is thought to be rapid, forceful descent of the fetal head into the birth canal and wedging of the head against the anterior pelvic ring, creating mechanical shear and ligament rupture. Predisposition has been attributed to multiparity, complicated delivery, forceps or vacuum assisted delivery, shoulder dystocia, maternal hip dysplasia, or prior pelvic trauma [1,7]. In addition, hyperabduction of the thighs and epidural anesthesia also have been implicated [10]. Young in 1938 described a condition called "pelvic arthropathy of pregnancy" that involved symphyseal and sacroiliac injuries and believed the widening occurred throughout pregnancy, not as an acute event during parturition [11]. Pain can be found over the SI joint and the inguinal area and in the deep pelvis and lumbar region. The patient may be unable to stand, stooping is performed with difficulty, and a waddling gait ensues. Trendelenburg's sign may be present [1,8].

Treatment of postpartum symphyseal rupture has traditionally been non-operative. Historically, bed rest, usually in a lateral decubitus position, analgesics, and the application of a pelvic binder to facilitate reduction of the diastasis are routinely sufficient to ensure full recovery in most case. Close follow-up is imperative to be certain of the effectiveness of non-operative therapy. Recovery from symphyseal rupture can be expected within 6 weeks. Operative treatment of the postpartum unstable pelvis has been advocated rarely and should be considered if conservative treatment has failed to control symptoms of severe pain [8]. The successful surgical treatment of the chronic postpartum pelvic pain usually is anterior pubic fixation with or without SI joint stabilization. Hagen in 1974 reported on 23 patients with "pelvic girdle relaxation"; eight of these patients were treated operatively, two with symphyseodesis, four with sacroiliac arthrodesis, and two with a combination of both procedures [8]. He recommended that operative therapy be considered in the patient with pubic diastasis of more than 1 cm, symphyseal shift of more than 5 cm, widening and sclerosis of the sacroiliac joints. Rommens presented three patients with postpartum symphysis pubis rupture whose severe complaints persisted after conservative treatment, two with 1.5 cm diastasis, and one with 4.0 cm diastasis. All three ruptures were stabilized and achieved a full recovery with open reduction and internal fixation [12]. Kharrazi et al also reported poor outcome in four cases with conservative treatment, and suggested non-operative treatment with bed rest and a properly positioned pelvic binder in patients with a symphyseal diastasis of less than 4.0 cm and a formal examination of symphyseal and sacroiliac instability under anesthesia, followed by anterior plate fixation for patients with more than 4.0 cm symphyseal widening [7]. Hierholzer et al reported one case with 9 cm postpartum symphysis pubis rupture successfully treated by anterior pubic symphysiodesis with two side SI joint arthrodesis [13]. These patients are challenged by the needs of their newborn child and family. Therefore, prolonged bed rest must be balanced with the risks of surgical intervention.

Various methods of operative fixation are available to stabilize pubic diastasis including anterior cerclage wiring, anterior plating and external fixation. Anterior plating can achieve superior reduction and healing rates compared with non-operative methods and external fixation [14,15]. In most cases, single-plate fixation of the pubic symphysis is sufficient. However, the addition of a second orthogonal plate may yield a stiffer construct and theoretically can stand earlier weight bearing in this severe disruption. Surgical intervention, sometimes, should be designated to hasten mobility and weight bearing.

Tile and Pennal described the use of orthogonal plates for fixation of the pubic symphysis significantly increased pelvic ring stability, one on the superior surface and one on the anterior surface [16]. Several biomechanical studies demonstrated dual plating resulted in less symphyseal displacement and improved posterior stability in simulated vertically unstable pelvic disruptions [17-19]. In first case, for the reason of severe instability of the pelvic ring and need for earlier weight bearing, we placed two orthogonal plates. After that, examination of the sacroiliac joint found posterior subluxation, therefore a sacroiliac screw was placed to stabilize the sacroiliac joint.

## Conclusions

Postpartum disruption of the pubic symphysis should be evaluated carefully with regards to the injury and the specific needs of the individual patient. Symptomatic wide disruption that does not decrease significantly in the six weeks may merit open reduction and internal fixation. Initial observation and repeat examination can help determine the best treatment for an individual patient.

## Consent

Written informed consents were obtained from the patients for publication of this two cases report.

## Competing interests

The authors declare that they have no competing interests.

## Authors' contributions

All authors contributed equally to this case report. All authors read and approved the final version of the manuscript.
